# A Systematic Review with Meta-Analysis of Comparative Efficacy and Safety of Risankizumab and Ustekinumab for Psoriasis Treatment

**DOI:** 10.1155/2022/2802892

**Published:** 2022-08-18

**Authors:** Qianying Yu, Xiaopei Ge, Mingyi Jing, Xiongfei Mi, Jing Guo, Min Xiao, Qing Lei, Mingling Chen

**Affiliations:** Department of Dermatology, Hospital of Chengdu University of Traditional Chinese Medicine, Chengdu, 610075 Sichuan Province, China

## Abstract

Biological targeted therapy serves as a new alternative treatment for psoriasis due to its minimal side effects. This study is aimed at examining the drug effectiveness and safety of risankizumab and ustekinumab for psoriasis treatment, so as to provide a reference for clinical decision-making. Databases from Embase, Web of Science, PubMed, and Cochrane Library were gathered, starting from inception to March 1, 2022, for randomized controlled trials regarding risankizumab and ustekinumab for psoriasis treatment. All retrieved articles were carefully selected in strict accordance with a set of inclusion and exclusion criteria. Stata 15.0 and RevMan 5.4 were applied to perform meta-analysis and risk of bias assessment. A total of two trials with three NCTs were selected, with 384 participants in the risankizumab group and 140 participants in ustekinumab. Meta-analysis showed that in the long-term and short-term PASI100, risankizumab was more effective than ustekinumab (RR = 2.27, 95% CI (1.77, 2.90), *p* < 0.05; RR = 2.33, 95% CI (1.75, 3.08), *p* < 0.05). In PASI90, RR = 1.77, 95% CI (1.54, 2.03), and *p* < 0.05 and RR = 1.72, 95% CI (1.48, 2.00), and *p* < 0.05. In short-term PASI75, RR = 1.23, 95% CI (1.13, 1.34), and *p* < 0.05. In sPGA of 0, the results at week-16 and week-52 showed that risankizumab was significantly more effective than ustekinumab (RR = 2.24, 95% CI (1.67, 3.01), *p* < 0.05; RR = 2.30, 95% CI (1.80, 2.95), *p* < 0.05). Risankizumab was significantly more effective than ustekinumab in improving the quality of life and PSS scores (RR = 1.48, 95% CI (1.26, 1.75), *p* < 0.05; RR = 2.01, 95% CI (1.41, 2.85), *p* < 0.05). Nevertheless, risankizumab and ustekinumab did not show significant difference in the incidence of adverse responses (RR = 1.02, 95% CI (0.75, 1.39), *p* > 0.05). Risankizumab was more effective than ustekinumab for the treatment of psoriasis. The adverse reactions of both risankizumab and ustekinumab were similar and could be tolerated. Risankizumab might be a better alternative option for their treatment.

## 1. Introduction

Psoriasis is a prevalent chronic inflammatory dermatosis with a high recurrence rate and is associated with abnormal autoimmunity. In 2014, WHO defined it as a chronic, noninfectious, painful, disfiguring, and disabling disease that can hardly be cured [1]. The global morbidity of psoriasis is 19 percent [2], with uneven distribution in different geographic regions. The incidence of psoriasis is similar between males and females, with the average age of onset of 33 years old, and approximately one-third of the patients have a family history [3]. Most psoriatic patients have compromised quality of life, and virtually, the most important source of stress for them in daily life is that they intend to avoid the mental impact caused by extensive lesions that might involve critical parts of the body like their face and palms. Psoriatic patients are more susceptible to depression (20%), compared with the healthy population, and are probable to commit suicide [4–6]. It is a long-term disease, with currently no effective therapeutic approaches [7]. Treatment for psoriasis mainly includes topical drug therapy, physiotherapy, and systemic drug therapy, and the former two are recommended for patients with mild symptoms while the latter is for moderate to severe patients. However, conventional drug therapy is often accompanied by several problems such as poor therapeutic effects, side effects, and hyporesponsiveness, which makes it urgent to explore a new therapeutic approach [8, 9].

An abnormal immune response is essential in the pathogenesis of psoriasis, including T-lymphocyte activation, leukocyte extravasation, and generation and release of multiple cytokines, and the biological agents alleviate the disease by suppressing the T lymphocyte-mediated immune response. It is generally accepted that interleukin-23 (IL-23)/interleukin-17A (IL-17A) axis plays a pivotal role in the psoriasis etiology. Members of interleukins that can be used as the pharmacological targets for psoriasis are primarily IL-12, IL-17, and IL-23 [10]. Ustekinumab, an IL-12/23 inhibitor, can specifically bind to the p40 subunit that exists in both IL-12 and IL-23 and subsequently block the binding of IL-12 and IL-23 to the receptors to reduce their activity, so as to alleviate the inflammatory response [11]. Risankizumab can suppress the release of proinflammatory cytokines and chemokines through binding to the p19 subunit of IL-23, so as to alleviate inflammation and attenuate symptoms of psoriatic patients [11–14]. It is quite controversial whether ustekinumab or risankizumab should be applied in the treatment of psoriasis; therefore, the aim of this study is to critically evaluate the efficacy and safety of ustekinumab and risankizumab in psoriasis treatment and provide a reference for the clinical decision-making.

## 2. Methods and Materials

This meta-analysis strictly abided by the PRISMA statement and was registered on INPLASY (INPLASY protocol 202270021) [15].

### 2.1. Literature Search

Databases from different sources such as Embase, PubMed, Cochrane Library, and Web of Science were obtained, from inception to March 1, 2022, to identify clinical trials regarding ustekinumab and risankizumab for the treatment of psoriasis. Search items included “Ustekinumab”, “Risankizumab”, “psoriasis”, “Psoriases”, and “PalmoplantarisPustulosis”. There is no restriction for publication data, language, and regions. PubMed search strategy is shown in Supplement file 1.

### 2.2. Inclusion Criteria

In the research subject or patients who meet the diagnostic criteria [16] of psoriasis and receive ustekinumab or risankizumab as an intervention, the primary outcomes included Psoriasis Area and Severity Index (PASI), a static Physician's Global Assessment (sPGA), quality of life, and psoriasis severity scale (PSS). The secondary outcome is the adverse events (AEs); this study used a randomized controlled trial (RCT) design.

### 2.3. Exclusion Criteria

Exclusion criteria are as follows: inapplicable study design, abstract of conference, repeated publication, letter, animal study, full-text unavailable, and necessary data unavailable.

### 2.4. Data Extraction

Article selection and data extraction were performed by two reviewers independently. All related articles were imported in EndNote (version 20) to rule out the duplicated, then screened through reading titles and abstracts, and finally screened through full-text reading to identify eligible studies.

The basic characteristics were extracted by two reviewers independently, which primarily included NCT number, publication data, nation, intervention, sample size, age of participants, outcome measures, and follow-up.

### 2.5. Quality Assessment

In order to examine the risk of bias, the Cochrane risk of bias assessment tool was applied for every included RCT, which refers to the following aspects: randomizing sequence generation, allocation concealment, blinding for patients, intervention givers, outcome measures, incomplete data, selective reporting, and other sources of bias. The assessment risk of bias for each aspect was categorized as low, high, or unclear [17].

### 2.6. Statistical Analysis

Data were pooled using the Stata 15.0 software. The mean difference (MD) with 95% confidence interval (95% CI) was used as a pooled statistic for continuous data, and the risk ratio (RR) with 95% CI was used for dichotomous data, with a *p* value less than 0.05 being considered statistically significant. *Q* test (*p* ≥ 0.1) and chi-square test (*I*^2^ ≤ 50%) would indicate less possibility of heterogeneity; then, the fixed-effect model would be applied for data synthesis; otherwise, high probability of heterogeneity would be considered, and a random-effect model should be applied, or the heterogeneity source would be identified via a subgroup analysis. Egger's test was used to assess publication bias in which *p* > 0.05 indicated a low chance of publication bias.

## 3. Results

### 3.1. Literature Screening

**Figure 1:**
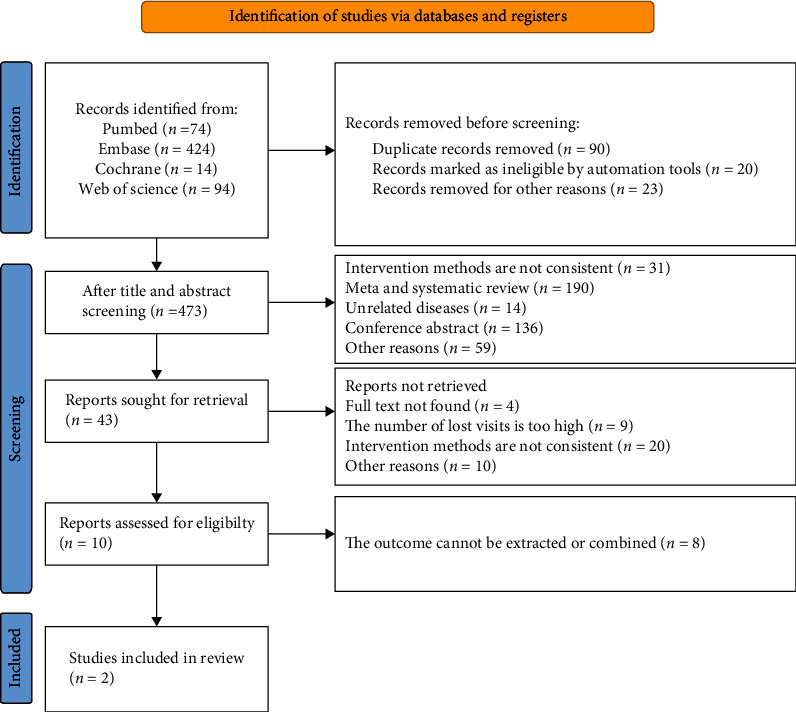
Flow diagram of literature search.

Through the first screening of PubMed, Embase, and Cochrane Library databases, 606 articles were identified, and 473 were retrieved after checking the duplicates. Through reading titles and abstracts, 43 articles remained, and 2 articles [18, 19] of high quality were included after full-text reading, which contains 3 NCTs. The schematic of the literature screening is illustrated in Figure 1.

### 3.2. Main Features of Included Studies

Two RCTs of high quality were included in this study, and one [19] of which contains 2 NCTs. All of the trials used risankizumab as the experimental group (681 participants) and ustekinumab as the control group (243 participants). Most of the participants were middle-aged males, and the follow-up ranged from 12 to 52 weeks. The main features of included studies are illustrated in Table 1.

### 3.3. Quality Assessment for Included Studies

**Figure 2:**
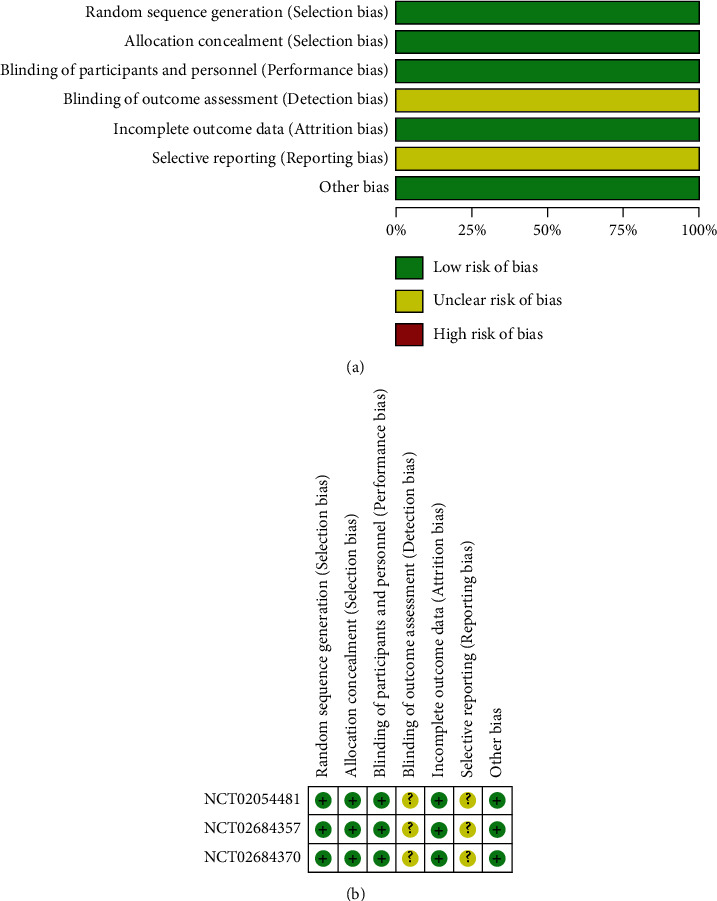
(a, b) Risk of bias of included studies.

The Cochrane risk of bias assessment tool was used to evaluate the risk of bias in the included studies. All the included studies were of high quality, as shown in Figures 2(a) and 2(b).

## 4. Results of Meta-analysis

### 4.1. Long-Term Scores of PASI (Psoriasis Area and Severity Index)

**Figure 3:**
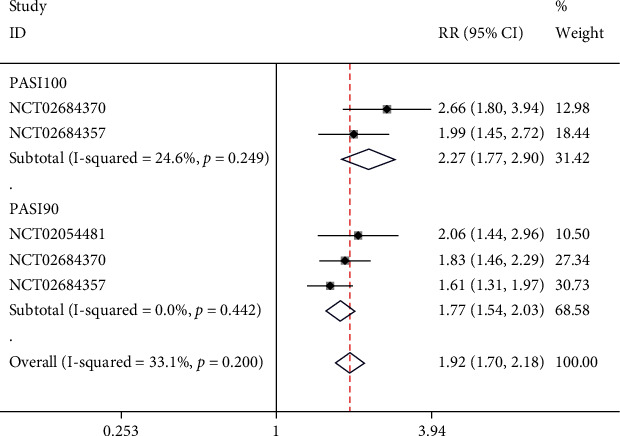
Forest plot of long-term PASI scores.

All the two included studies [18, 19] reported the long-term scores of PASI, with 3 NCTs included in the analysis. Subgroups were set based on PASI scores. There was no significant heterogeneity in PASI100 subgroup (*I*^2^ = 24.6%, *p* = 0.249); thus, a fixed-effect model was employed. The result of meta-analysis indicated that in PASI100, risankizumab was significantly more effective than ustekinumab (RR = 2.27, 95% CI (1.77, 2.90), *p* < 0.05). Furthermore, the heterogeneity in PASI 90 subgroup was not significant (*I*^2^ = 0%, *p* = 0.442); hence, a fixed-effect model was used. According to the meta-analysis result, risankizumab was significantly more successful than ustekinumab in PASI90 (RR = 1.77, 95% CI (1.54, 2.03), *p* < 0.05), as illustrated in Figure 3.

### 4.2. Short-Term Scores of PASI

**Figure 4:**
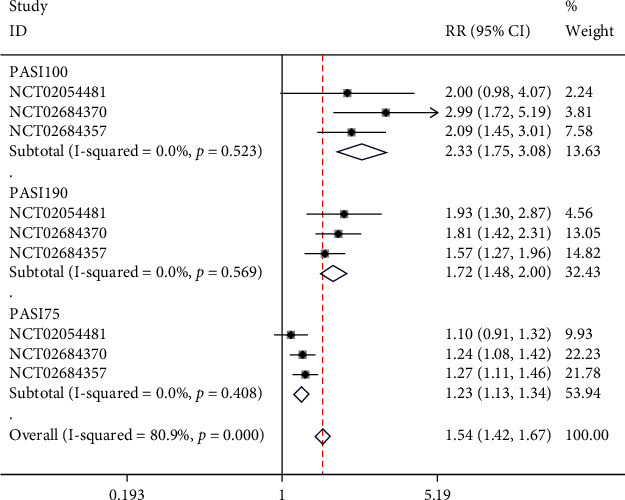
Forest plot of short-term PASI scores.

All the two included studies [18, 19] reported the short-term scores of PASI, with 3 NCTs included in the analysis. Subgroups were set based on PASI scores. There was no significant heterogeneity in PASI100 subgroup (*I*^2^ = 0%, *p* = 0.523); hence, fixed-effect model was employed. Furthermore, risankizumab showed more efficacious than ustekinumab according to the meta-analysis in PASI100 (RR = 2.33, 95% CI (1.75, 3.08), *p* < 0.05). Furthermore, heterogeneity in the PASI 90 subgroup was not significant (*I*^2^ = 0 percent, *p* = 0.569); thus, a fixed-effect model was applied. The results suggested that in PASI90, risankizumab had a statistically significant therapeutic benefit over ustekinumab (RR = 1.72, 95% CI (1.48, 2.00), *p* < 0.05). There was no significant heterogeneity in PASI75 subgroup (*I*^2^ = 0%, *p* = 0.569); hence, a fixed-effect model was used. The results demonstrated that in PASI75, risankizumab was significantly more effective than ustekinumab (RR = 1.23, 95% CI (1.13, 1.34), *p* < 0.05), as illustrated in Figure 4.

### 4.3. Long-Term Scores of sPGA (Static Physician's Global Assessment)

**Figure 5:**
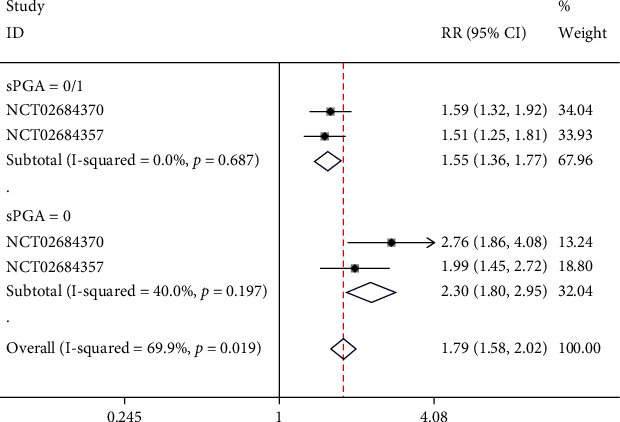
Forest plot of long-term sPGA scores.

One study containing 2 NCTs reported sPGA. Subgroups were set based on the scores of sPGA. In the subgroup with a sPGA score of 0/1, no significant heterogeneity was found (*I*^2^ = 0%, *p* = 0.687), and a fixed-effect model was employed. The results showed that in sPGA 0/1, risankizumab was significantly more effective than ustekinumab (RR = 1.55, 95% CI (1.36, 1.77), *p* < 0.05). In the subgroup with ansPGA score of 0, no significant heterogeneity was found (*I*^2^ = 40%, *p* = 0.197), and a fixed-effect model was employed. The results showed that in sPGA 0, risankizumab was significantly more effective than ustekinumab (RR = 2.30, 95% CI (1.80, 2.95), *p* < 0.05), as depicted in Figure 5.

### 4.4. Short-Term Scores of sPGA

**Figure 6:**
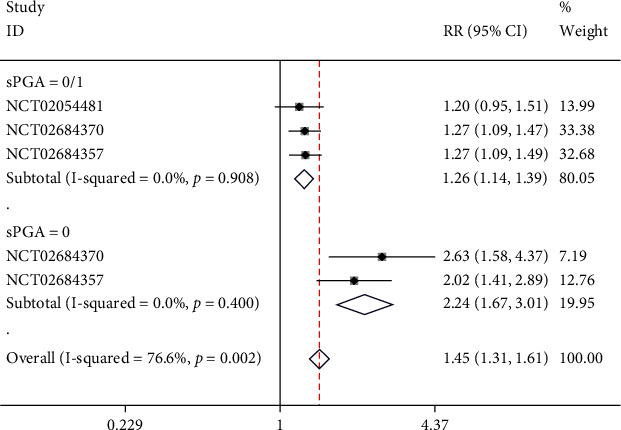
Forest plot of short-term sPGA scores.

One study containing 2 NCTs reported sPGA. Subgroups were set based on the scores of sPGA. In the subgroup with a sPGA score of 0/1, no significant heterogeneity was found (*I*^2^ = 0%, *p* = 0.908); thus, a fixed-effect model was employed. Meta-analysis indicated that in sPGA 0/1, risankizumab was significantly more effective than ustekinumab (RR = 1.26, 95% CI (1.16, 1.39), *p* < 0.05). In the subgroup with a sPGA score of 0, no significant heterogeneity was found (*I*^2^ = 0%, *p* = 0.400), and a fixed-effect model was applied. The meta-analysis result showed that in sPGA 0, risankizumab was significantly more effective than ustekinumab (RR = 2.24, 95% CI (1.67, 3.01), *p* < 0.05), as illustrated in Figure 6.

### 4.5. sPGA Score of 0/1

**Figure 7:**
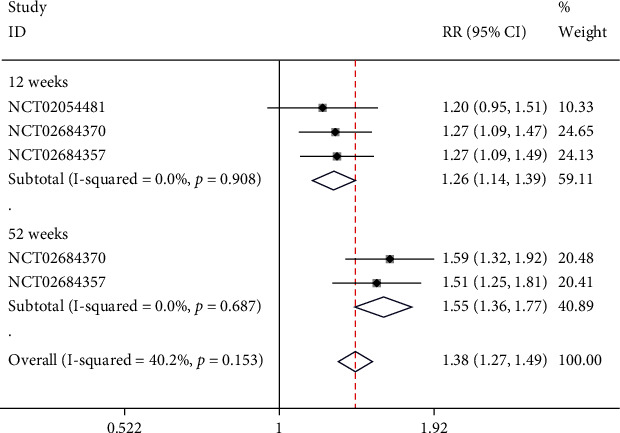
Forest plot of sPGA scores of 0/1.

Two studies including 3 NCTs reported sPGA. Subgroups were set based on the time of scoring. In the week-12 subgroup, there was no significant heterogeneity observed (*I*^2^ = 0%, *p* = 0.908); thus, a fixed-effect model was applied. Moreover, the score of sPGA in week-12 was 0/1, and risankizumab was significantly more effective than ustekinumab (RR = 1.26, 95% CI (1.34, 1.39), *p* < 0.05). In week-52 subgroup, no significant heterogeneity was noticed (*I*^2^ = 0%, *p* = 0.908), and a fixed-effect model was used. The meta-analysis demonstrated that the score of sPGA in week-52 was 0/1 and risankizumab was significantly more effective than ustekinumab (RR = 1.55, 95% CI (1.36, 1.77), *p* < 0.05), as illustrated in Figure 7.

### 4.6. sPGA Score of 0

**Figure 8:**
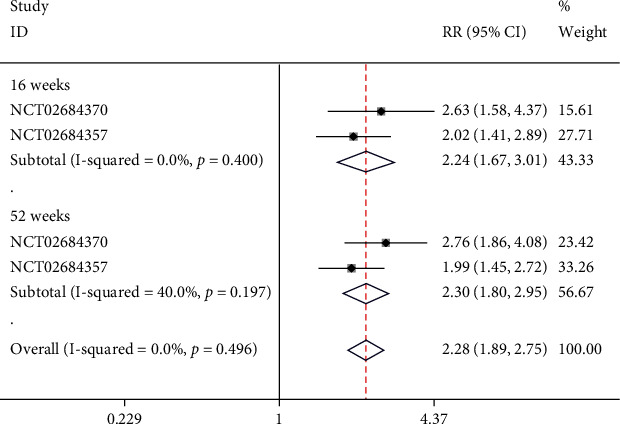
Forest plot of sPGA scores of 0.

There was one study including 2 NCTs that reported sPGA. Subgroups were set based on the time of scoring. In the week-16 subgroup, no significant heterogeneity was observed (*I*^2^ = 0%, *p* = 0.400), and a fixed-effect model was employed. The score of sPGA in week-16 was 0, and risankizumab was significantly more effective than ustekinumab (RR = 2.24, 95% CI (1.67, 3.01), *p* < 0.05). In week-52 subgroup, no significant heterogeneity was noticed (*I*^2^ = 40%, *p* = 0.197); thus, a fixed-effect model was used. The result of meta-analysis demonstrated that the score of sPGA in week-52 was 0 and risankizumab was significantly more effective than ustekinumab (RR = 2.30, 95% CI (1.80, 2.95), *p* < 0.05), as illustrated in Figure 8.

### 4.7. Psoriasis Severity Scale (PSS)

**Figure 9:**
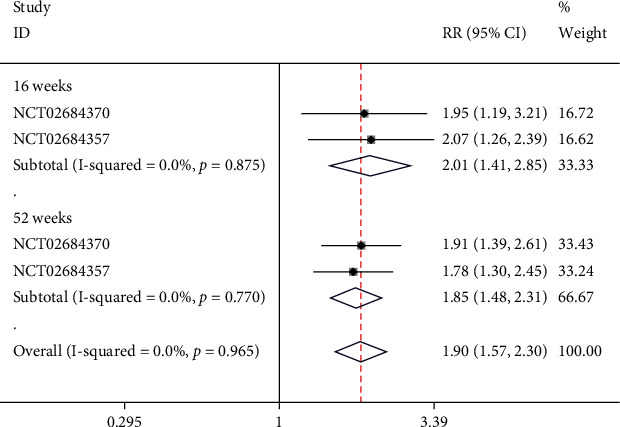
Forest plot of PSS.

There was 1 study containing 2 NCTs that reported PSS. Subgroups were set based on the time of scoring. In the week-16 subgroup, no significant heterogeneity was found (*I*^2^ = 0%, *p* = 0.875), and a fixed-effect model was employed. Moreover, the score of PSS in week-16 was significantly better in the risankizumab group than in the ustekinumab group (RR = 2.01, 95% CI (1.41, 2.85), *p* < 0.05). In the week-52 subgroup, no significant heterogeneity was found (*I*^2^ = 0%, *p* = 0.770), and a fixed-effect model was applied. Meta-analysis showed that the score of PSS in week-52 was significantly better in the risankizumab group than in the ustekinumab group (RR = 1.85, 95% CI (1.48, 2.31), *p* < 0.05), as illustrated in Figure 9.

### 4.8. Quality of Life

**Figure 10:**
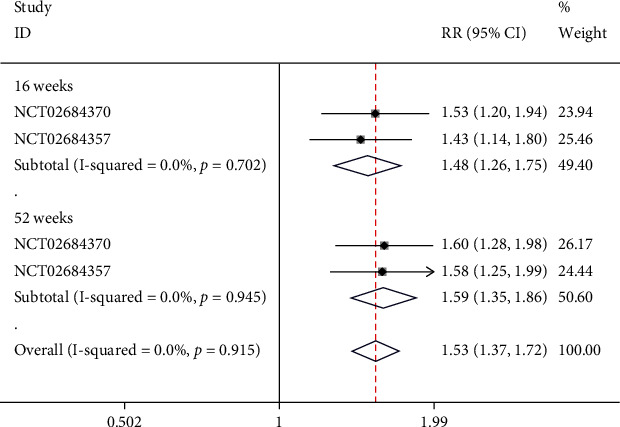
Forest plot of quality of life.

There was one study containing 2 NCTs that reported the quality of life indices. Subgroups were set based on the follow-up periods. In the week-16 subgroup, no significant heterogeneity was found (*I*^2^ = 0%, *p* = 0.702), and a fixed-effect model was employed. The results indicated that the improvement in quality of life in week-16 was significantly greater in the risankizumab group than in the ustekinumab group (RR = 1.48, 95% CI (1.26, 1.75), *p* < 0.05). In the week-52 subgroup, no significant heterogeneity was found (*I*^2^ = 0%, *p* = 0.945), and a fixed-effect model was used. The results indicated that the improvement in quality of life in week-52 was significantly greater in the risankizumab group than in the ustekinumab group (RR = 1.59, 95% CI (1.35, 1.86), *p* < 0.05), as illustrated in Figure 10.

### 4.9. Adverse Reactions

**Figure 11:**
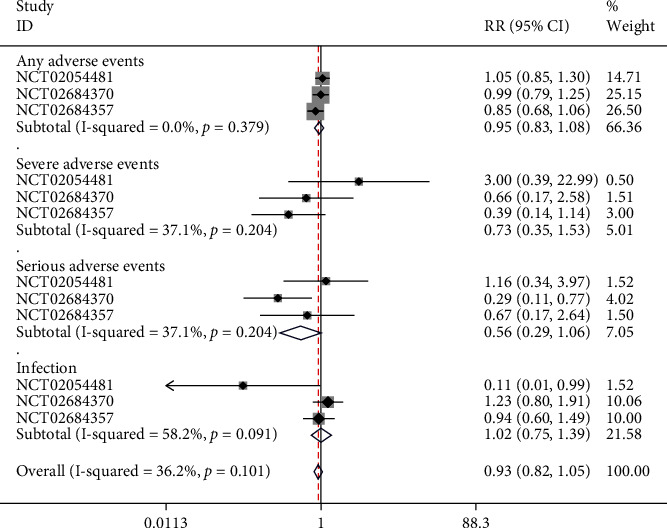
Forest plot of adverse reactions.

All the two studies containing 3 NCTs reported adverse events, and the subgroups were set based on different types of adverse events. In the subgroup of any adverse reaction, no significant heterogeneity was found (*I*^2^ = 0%, *p* = 0.379), and a fixed-effect model was used. It was found that the frequency of any adverse event between the risankizumab and ustekinumab groups did not differ significantly (RR = 0.95, 95% CI (0.83, 10.8), *p* > 0.05). In the subgroup of severe adverse reaction, no significant heterogeneity was found (*I*^2^ = 37.1%, *p* = 0.204), and a fixed-effect model was applied. The results showed that there was no significant difference in the incidence of severe adverse reactions between risankizumab group and ustekinumab group (RR = 0.73, 95% CI (0.35, 1.53), *p* > 0.05). In the subgroup of serious adverse reaction, no significant heterogeneity was found (*I*^2^ = 37.1%, *p* = 0.204), and fixed-effect model was applied. The result showed that the incidence of serious adverse reactions between risankizumab group and ustekinumab group was not significantly different (RR = 1.02, 95% CI (0.75, 1.39), *p* > 0.05), as illustrated in Figure 11.

### 4.10. Sensitivity Analysis and Publication Bias Assessment

All indices analyzed in this study have heterogeneity of less than 50%. In sensitivity analysis, the results did not reverse after the removal of a certain result, and the results of the two effect models were similar. On the other hand, publication bias could not be conducted due to the limited studies included.

## 5. Discussion

Psoriasis is a chronic, recurrent, and systemic disease commonly seen in the dermatological department, which could not be completely cured to this day [20]. The selection of therapeutic approaches should not only take into account patients' conditions like the severity of disease and concomitant joint damage but also the long-term therapeutic effects, patients' compliance, tolerance, etc. Conventional systematic treatments for psoriasis include methotrexate, cyclosporine, acitretin, and phototherapy, while the long-term use of these agents is limited due to safety concerns about the potential hepatotoxicity, renal toxicity, and phototoxicity [21–24]. In the past decades, innovative approaches including biological agents have emerged and been wildly applied, such as TNF-*α* inhibitors that have been approved in China for the treatment of psoriasis. IL-23, IL-12, and IL-17 have increasingly become new target molecules as research has progressed [25]. Studies have demonstrated that the IL-23 and IL-12 receptor antagonists, risankizumab and ustekinumab, could improve patients' well-being with psoriasis and the main outcomes for the assessment of the clinical effects include PASI and Dermatology Life Quality Index (DLQI) [26–28]. LOTUS [29], a phase-III clinical trial with 322 Chinese participants, is aimed at evaluating the effect of ustekinumab on moderate to severe psoriatic patients, and the results suggested that at week-12, the PASI75 reaction in patients receiving ustekinumab 45 mg (82.5%) was significantly higher than those who received placebo (11.1%). After 12 weeks, the PASI7 response rate in ustekinumab group continued to increase and peaked at week-24 (91.6%). The good response could be maintained until the end of the trial (91.5%).

Furthermore, the study revealed that the number of participants in PAS100, PAS90, and PAS75, either in the short-term or the long-term follow-up, was greater in the risankizumab group than in the ustekinumab group, suggesting that risankizumab was more effective than ustekinumab for psoriasis treatment, which was similar to results of a previous study conducted by Bai et al. [30] In sPGA of 0 and 0/1, the number of participants in the risankizumab group was greater than that in the ustekinumab group during the follow-up from week-16 to week-52, which also indicated better effect of risankizumab. As for the improvements in quality of life and PSS scores, risankizumab was more effective than ustekinumab for psoriasis treatment. Reich et al. [31] reported that risankizumab treatment resulted in higher psoriatic lesion clearance (*p* < 0.05), and the effect was lasting. Moreover, it was found that there was no significant difference in the frequency of severe adverse responses, any adverse reactions, or infection between risankizumab and ustekinumab, indicating that the two medicines were similarly safe. Studies by Leonardi et al. [32] and Papp et al. [18] demonstrated that the effect appeared 2 weeks after the first intravenous administration of ustekinumab and would last for one and a half years after being treated once every 12 weeks, while the disease relapsed gradually after the drug discontinuance accompanying with temporary IL-12/IL-23 resistance, which suggested that maintaining treatment is required for the lasting therapeutic effect. Retreatment would take effect after 12 weeks.

This study included 2 studies containing 3 NCTs which all of them are randomized, double-blind, and multicenter trials of high quality. Though the overall quality was considerable, there are some limitations:

1. Few studies are included, with limited sample size. More positive-controlled are required to comprehensively evaluate the effect and safety of risankizumab and ustekinumab drugs
2. The follow-up periods are short. There is a need for more well-designed multicenter RCTs with big sample sizes and long-term follow-up to identify rare adverse events and adverse events with long incubation periods like malignancy
3. The safety evaluation only included the results in 16 weeks; thus, potential rare AEs or AEs that take a long time to be recognized could not be detected. The results of this study should be considered prudently in combination with experience and clinical practice

## 6. Conclusion

To sum up, risankizumab was more effective than ustekinumab for the treatment of psoriasis. The adverse reactions of both risankizumab and ustekinumab are the same and can be tolerated. Risankizumab might be a new option for their treatment.

## Figures and Tables

**Table 1:** Basic characteristics of included studies.

First author	Publication date	NCT number	Nation	Study design	Intervention		Sample size (male)		Age		Outcomes	Follow-up (week)
					EG	CG	EG	CG	EG	CG		
Kim A Papp	2017	NCT02054481	USA	RCT	Risankizumab 90 mg 180 mg	Ustekinumab 45 mg weight ≤ 100 kg 90 mg weight > 100 kg	41 (30) 42 (29)	40 (27)	49 ± 13 45 ± 14	45 ± 12	F1, F2, F3	12
Kenneth B Gordon	2018	NCT02684370 (UltIMMa-1) NCT02684357 (UltIMMa-2)	USA	RCT	Risankizumab UltIMMa-1 150 mg UltIMMa-2 150 mg	Ustekinumab UltIMMa-1 45 or 90 mg UltIMMa-2 45 or 90 mg	304 (212) (UltIMMa-1) 294 (203) (UltIMMa-2)	100 (70) (UltIMMa-1) 99 (66) (UltIMMa-2)	48.3 ± 13.4 (UltIMMa-1) 46.2 ± 13.7 (UltIMMa-2)	46.5 ± 13.4 (UltIMMa-1) 48.6 ± 14.8 (UltIMMa-2)	F1, F2, F3, F4, F5	52

RCT: randomized controlled trial; EG: experimental group; CG: control group; F1: Psoriasis Area and Severity Index (PASI); F2: adverse events; F3: a static Physician's Global Assessment (sPGA); F4: quality of life; F5: PS.

## Data Availability

Data sharing is not applicable to this article as no datasets were generated or analyzed during the current study.

## Supplementary Materials

Supplementary Materials Supplementary file 1: search strategies in PubMed.
