# Supplementary material for: A Systematic Review with Meta-Analysis of Comparative Efficacy and Safety of Risankizumab and Ustekinumab for Psoriasis Treatment
Source: J Immunol Res. 2022 Aug 18;2022:2802892. doi: 10.1155/2022/2802892 (PMC9410857; doi:10.1155/2022/2802892)
Supplement: Supplementary Materials — Supplementary file 1: search strategies in PubMed. [file 2802892.f1.docx]

**Supplementary file 1:** Search strategies in PubMed

| Search strategies in PubMed. | |
| --- | --- |
| ID | Query |
| #1 | "Psoriasis"[Mesh] |
| #2 | (((((Psoriasis[Title/Abstract]) OR (Psoriases[Title/Abstract])) OR (Pustulosis of Palms[Title/Abstract] AND Soles[Title/Abstract])) OR (Pustulosis Palmaris et Plantaris[Title/Abstract])) OR (Palmoplantaris Pustulosis[Title/Abstract])) OR ("Pustular Psoriasis of Palms and Soles"[Title/Abstract]) |
| #3 | #1 OR #2 |
| #4 | "risankizumab" [Supplementary Concept] |
| #5 | (((((risankizumab[Title/Abstract]) OR (ABBV-066[Title/Abstract])) OR (skyrizi[Title/Abstract])) OR (risankizumab-rzaa[Title/Abstract])) OR (BI 655066[Title/Abstract])) OR (BI-655066[Title/Abstract]) |
| #6 | #4 OR #5 |
| #7 | "Ustekinumab"[Mesh] |
| #8 | (((Ustekinumab[Title/Abstract]) OR (Stelara[Title/Abstract])) OR (CNTO 1275[Title/Abstract])) OR (CNTO-1275[Title/Abstract]) |
| #9 | #7 OR #8 |
| #10 | #3 AND #6 AND #9 |
